# Design of a Game-Based Training Environment to Enhance Mental Health Care Professionals’ Skills in Using e–Mental Health: Multiple Methods User Requirements Analysis

**DOI:** 10.2196/34700

**Published:** 2022-07-27

**Authors:** Joyce Bierbooms, Milou A Feijt, Wijnand A IJsselsteijn, Inge M B Bongers

**Affiliations:** 1 Tranzo Tilburg School of Social and Behavioral Sciences Tilburg University Tilburg Netherlands; 2 Human-Technology Interaction Eindhoven University of Technology Eindhoven Netherlands; 3 GGz Eindhoven en de Kempen Eindhoven Netherlands

**Keywords:** serious gaming, e–mental health, mental health care, skill development, game design, user requirements, mobile phone

## Abstract

**Background:**

A major factor hampering the adoption of technology in mental health care is a lack of knowledge and skills. Serious gaming offers a potentially effective strategy to enhance the skills needed through experiencing and learning-by-doing in a playful way. However, serious gaming solutions are not widely available for mental health care. Therefore, the development of a game-based training environment in mental health care was pursued in a design project. The first step in such a design project is to identify user requirements that should be met.

**Objective:**

This study aims to deliver user requirements that inform the design of a game-based training environment for mental health care professionals. This environment aims to support mental health care professionals’ knowledge and skill enhancement regarding the use of e–mental health (eMH); for example, video calling, mobile apps, web-based treatment modules, and techniques such as virtual or augmented reality.

**Methods:**

We used an exploratory multiple methods design consisting of a web-based questionnaire, co-design sessions, and interviews. To ensure a good representation of the target user group, professionals from various disciplines within mental health care were included in the research. The multiple methods design facilitates a broad view of user needs and in-depth knowledge of specific design requirements. We describe the protocol for this research project in a protocol paper published in the *JMIR Research Protocols* in February 2021.

**Results:**

The user requirements analysis revealed three types of users for the envisioned game-based training environment: mental health care professionals who want to learn about the basic possibilities of eMH, mental health care professionals who want to develop their eMH skills to the next level, and mental health care professionals who want to experiment with new technologies. This reflects the diversity of needs that were identified, as well as the need to develop a diversity of suitable scenarios in the environment. User requirements analysis shows that the focus of a training environment should be on increasing knowledge about the possibilities of eMH, focusing on experiencing the benefits in particular situations, and building confidence in using eMH in a therapeutic setting. This requires careful consideration of the suitable game characteristics.

**Conclusions:**

Improvement of mental health care professionals’ skills in eMH requires an environment that is user driven and flexible, and simultaneously incorporates contextual factors that are relevant for its implementation in practice. This user requirements analysis contributes to the understanding of the issues that should be considered in the development of a game-based training environment. This shows that there are multiple and diverse learning needs among mental health care professionals. Various client populations, services, and situations demand various options for training.

**International Registered Report Identifier (IRRID):**

RR2-10.2196/18815

## Introduction

### Background

Although the rise of new technologies has offered numerous possibilities for new methods and means of treatment in mental health care, the uptake of such technologies by mental health care professionals is rather slow [1-3]. With such technologies, we refer to mental health services that are provided or supported by digital tools; for example, video calling, mobile apps for self-tracking or self-management, web-based treatment modules, and techniques such as virtual or augmented reality [4-6], hereafter referred to as e–mental health (eMH). One important factor hampering the adoption of eMH by mental health care professionals is a lack of knowledge and skills of mental health care professionals to effectively find and use web-based technologies and their corresponding need to gain more experience in this [7-10]. This knowledge and skill gap concerns different types of knowledge and skills regarding the use of eMH [8,11]. For example, a lack of skills may exist regarding the ability to properly use technological devices or regarding knowledge about the availability of eMH tools that can be applied for different therapies. Another example is the perceived difficulties in establishing rapport in remote forms of communication [8,11].

### Skill Enhancement Through Game-Based Training

Multiple training concepts have been developed in mental health care to enhance professionals’ skills in using eMH, such as classical instructions, individual e-learning, and demonstrations. However, they have not reached the point at which this has led to a substantial expansion of knowledge and skills regarding eMH [12]. The reasons for this are the lack of interactivity and experiential learning in these concepts [13]. Therefore, a new potential strategy to address this issue may be to offer mental health care professionals training possibilities in a game-based environment, where skill enhancement is achieved through experiencing and learning-by-doing in a playful way [13]. Multiple positive outcomes of game-based learning have already been demonstrated in other areas of business [14], and positive effects have been reported in several studies on serious gaming to train health care professionals [13]. A major advantage of serious gaming lies in the fact that it offers a hands-on learning experience rather than just reading or hearing instructions [14,15]. It also offers a unique combination of education and fun [16]. Furthermore, serious games have demonstrated various learning outcomes (eg, cognitive skills, motor skills, affective learning outcomes, and communicative learning outcomes) that are more difficult to achieve through traditional learning methods [17]. These are strong arguments to believe that serious gaming is a potentially effective way to develop mental health care professionals’ skills in using eMH in an experience-based way. Moreover, it would be difficult, expensive, and unethical to experiment with real clients in this way [18,19]. Looking at the current literature on serious gaming, it can be noticed that such training possibilities have not yet been specifically designed for mental health care. Therefore, we intend to design a game-based training environment specifically for mental health care professionals in which they can explore eMH, practice with different technological tools, and enhance their knowledge and skills in eMH. Finally, this could stimulate the increasing use of eMH in daily practice.

To establish a game-based training environment that meets the specific needs of mental health care professionals, they should be involved from the start to inform designers of their needs and preferences. In this way, the design will reflect their actual work situations, the problems they encounter in these situations, and the needs and preferences regarding ways to tackle these problems [20-23]. The first step in such a design project is to identify the user requirements that the training environment should satisfy [24-27]. This means identifying and describing end users’ needs in terms of their characteristics, learning needs, and learning preferences. This information is used to develop scenarios that reflect the real-life situations of these end users and draw up a list of technical and organizational requirements that this game-based training environment should meet [12]. In this paper, we describe the results of the user requirements analysis that was conducted in light of the design of a game-based training environment for mental health care professionals in the Netherlands. This analysis aimed to gain a better understanding of mental health care professionals’ attitudes, skills, and ambitions regarding the use of eMH.

### Objectives

This study aimed to deliver user requirements that inform the design of a game-based training environment for mental health care professionals to enhance their knowledge and skills regarding the use of eMH [12]. The user requirements consist of (1) an elaborate analysis of end users’ (ie, mental health care professionals’) needs, (2) a description of possible scenarios regarding the content of a game-based training environment, and (3) the technical and organizational requirements that are expected to be critical for the successful implementation of a game-based training environment.

## Methods

### Overview

In this section, we provide a brief summary of the methods used, which are described in detail in a protocol study [12]. To extract user requirements, we used an exploratory multiple methods design consisting of a questionnaire, co-design sessions, and interviews. We used a web-based questionnaire (N=432) administered to various mental health care organizations to gather data on end users’ needs in terms of mental health care professionals’ characteristics, attitudes, and skill levels regarding eMH. A co-design session was organized with 9 mental health care professionals to elaborate on these characteristics, attitudes, and skill levels and to gain the first ideas about mental health care professionals’ learning preferences in a game-based training environment. For the data collection that informed the scenarios, another 2 co-design sessions were held, each with 10 participants, including mental health care professionals, innovations experts, developers, and researchers. Finally, we conducted 17 interviews with mental health care professionals. These interviews were aimed at learning about the preferences of mental health care professionals regarding training in eMH and the technical and organizational prerequisites for the envisioned game-based training environment. Textbox 1 presents an overview of the methods, describing the aim of each method and the participants involved.

**Overview of the aim and participants of each study method.**

**Web-based questionnaire**

- Aim
  Description of the characteristics of the user group
  Perceived problems related to e–mental health skills
- Participants
  N=432
  Male: 144 (33%)
  Female: 288 (67%)
  Average age: 41
  Professions
    Nursing professionals (32.4%, including specialized nursing)
    Psychologists (27.5%, also including specializations)
    Social workers (17.1%)
    Other (23%; for example, medical specialists, case managers, activity workers, and supporting staff)

**Co-design session 1**

- Aim
  Work context
  Perceived problems related to e–mental health skills
  Users’ preferences regarding a game-based training environment
- Participants
  N=9
  Male: 2 (22%)
  Female: 7 (78%)
  Professions
    Medical specialist (n=1, 11%)
    Psychologists (n=2, 22%)
    Nursing professionals or specialized nurses (n=4, 44%)
    Social workers (n=2, 22%)

**Co-design session 2**

- Aim
  Scenario development
- Participants
  N=10
  Male: 5 (50%)
  Female: 5 (50%)
  Professions
    Mental health care professionals (n=3, 30%)
    Game developers (n=3, 30%)
    Innovation experts (n=2, 20%)
    Researchers (n=2, 20%)

**Co-design session 3**

- Aim
  Scenario development
- Participants
  N=10
  Male: 5 (50%)
  Female: 5 (50%)
  Professions
    Mental health care professionals (n=3, 30%)
    Game developers (n=3, 30%)
    Innovation experts (n=2, 20%)
    Researchers (n=2, 20%)

**Interviews**

- Aim
  Users’ preferences regarding a game-based training environment
  Technical and organizational prerequisites
- Participants
  N=17
  Male: 4 (24%)
  Female: 13 (77%)
  Professions
    Medical specialist (n=1, 6%)
    Psychologists (n=3, 18%)
    (Specialized) nursing professionals (n=4, 24%)
    Social workers (n=3, 18%)
    Innovation staff (n=4, 24%)
    Management (n=2, 12%)

To ensure a good representation of the target user group for each data collection method, professionals from various disciplines involved in the direct care delivery process within mental health care were included in the research. Professionals of 5 large integrated mental health care organizations providing mental health care to clients with complex mental health problems participated in the questionnaire. Co-design sessions and interviews were conducted at one of these organizations (Stichting Geestelijke Gezondheidszorg Eindhoven en de Kempen [GGzE]) in the southern part of the Netherlands. The multiple methods design combines a broad view of user needs (through the web-based questionnaire) and in-depth knowledge of specific design requirements (through interviews and co-design). The questionnaire data were used to analyze, using descriptive statistics, which subgroups could be defined among mental health care professionals as potential users of eMH. In addition, the data were used to define the specific needs for each subgroup. The co-design sessions and interviews were analyzed using thematic coding and provided in-depth information on the requirements for a game-based training environment based on specific end user’s needs. The data were triangulated as described in the protocol paper [12].

### Ethics Approval

This study was approved by the ethical review board of Tilburg University (reference number: EC-2018.15) and the internal scientific review board of GGzE (reference number: MM/2019004).

## Results

### Overview

Following the approach described in the Methods section, this section presents the results of the user requirements analysis in the design process of a game-based training environment. First, we clarify the end users’ needs regarding a game-based training environment by providing: (1) a description of the characteristics of the user group (ie, mental health care professionals), (2) their work context and practices, (3) their perceived problems regarding the use of eMH, and (4) their preferences related to a game-based training environment. Subsequently, we describe the scenarios by describing how the added value of eMH tools can be effectuated in specific situations, combined with relevant workflows and processes, the use of specific eMH tools in different situations, and decisive moments regarding the use of eMH. Finally, we present the results of the study aimed at identifying the technical and organizational conditions that are needed to enable the use of the anticipated game-based training environment. This means that design- and content-related conditions as well as organizational stimuli that are required to launch a game-based training environment in practice were explored.

### End Users’ Needs

#### Description of the Characteristics of the User Group

On the basis of the survey data from mental health care professionals in the Netherlands, a number of general characteristics of the sample can be described. Most mental health care professionals were female (288/432, 66.8%), with an average age of 41.3 (SD 12.1) years, and many of them had been working as health care professionals for a relatively long period (average of 16.3 years, SD 11.4). Mental health care professionals were highly educated: 49.3% (213/432) of them have received higher vocational education and 38.7% (167/432) have an academic degree. The main professions that were found among the respondents of the survey were nursing professionals (140/432, 32.4%, including specialized nursing), psychologists (119/432, 27.5%, also including specializations), and social workers (74/432, 17.1%). Most mental health care professionals (278/432, 64.4%) indicated that they had not received specific eMH training.

To identify the extent to which mental health care professionals in the Netherlands have adopted eMH in their daily practice, the survey included a number of questions related to the adoption of eMH (Table 1). Only 26.3% (114/432) of mental health care professionals were active or innovative users of eMH.

**Table 1:** Levels of adoption of e–mental health (eMH) by mental health care professionals in the Netherlands (N=432).

Level of adoption of eMH	Values, n (%)
No use (“I do not really want to start using it”)	11 (25)
Minimal use (“It is not part of my daily routine”)	112 (26)
Passive use (“I use what is readily available”)	195 (45.2)
Active use (“I am exploring more possibilities”)	86 (19.8)
Innovative use (“I am going to build upon my new idea”)	28 (6.5)

#### Work Context

The first co-design session aimed to gather data to identify the work context of mental health care professionals. The data showed that mental health care professionals in the co-design session work with a client population with multiple complex mental health problems, including clients in a crisis situation, which means they work in a specialized mental health care setting. They worked in a clinical or ambulatory setting, the latter being the setting in which most of the respondents worked. Most ambulant treatment trajectories aimed to support clients during their recovery process. This means working toward a situation in which people are able to manage their own mental well-being and know how to cope with difficult situations to improve their quality of life. In many cases, mental health care professionals are also in contact with the family and friends of their clients, as well as organizations such as schools and financing institutions that are often involved in the treatment trajectories. Mental health care professionals state that it may be opportune to offer eMH options at some point during a treatment trajectory, for example, to keep contact with a client’s network or monitor a client’s functioning between treatment meetings.

#### Perceived Problems Related to eMH Skills

As mentioned in the Introduction section, one of the key problems for the large-scale adoption of eMH among mental health care professionals is the perceived lack of skills [7-10]. Insights from the survey provided an indication of how mental health care professionals perceive their skill levels regarding the use of eMH. Table 2 shows that a large percentage of mental health care professionals reported insufficient knowledge about the availability of eMH tools or lack knowledge on how to apply these tools. Of the people who are knowledgeable about how to use eMH to some extent, a large proportion only know the basics of a small number of tools.

**Table 2:** Self-perceived skill levels regarding the use of e–mental health (eMH) by mental health care professionals (N=432).

Self-perceived skill level	Values, n (%)
I have insufficient knowledge of what is available when it comes to eMH	104 (24.1)
I have an image of what eMH tools are available, but I do not know how to use these	74 (17.1)
I know how to use the basic functionalities of eMH	133 (30.8)
I am capable to explore the possibilities of eMH and how to use different tools	97 (22.5)
I feel very competent regarding many different eMH tools and I know how to share this expertise with others	24 (5.6)

When looking further into these skills, both the survey and the first co-design session revealed that different skill types play a role in using eMH. Most respondents indicated that they felt they had sufficient digital skills but lacked knowledge on how to communicate in a web-based contact with their clients. Moreover, the feeling of being unable to establish sufficient empathic understanding in a web-based conversation is an important issue for mental health care professionals. The types of skills that mental health care professionals feel they need to practice most, differ depending on their level of adoption of eMH. For example, the co-design session revealed that people who qualify themselves as minimal to passive users often want to find out more about what is actually available regarding eMH. People who are already active users want to take it a step further and want to discover new possibilities on how to create an empathic interaction.

**Figure 1:**
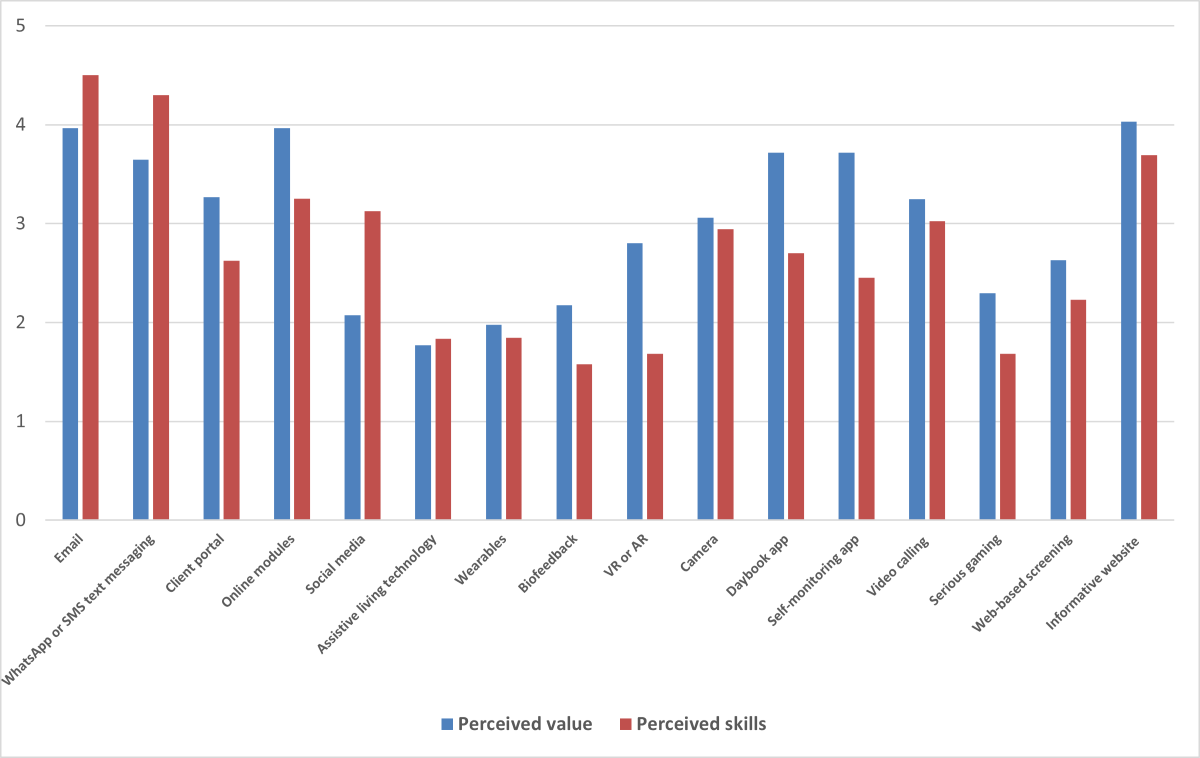
Perceived value and perceived skills of e–mental health tools. AR: augmented reality; VR: virtual reality.

The perceived skill levels of mental health care professionals also differ among the types of eMH tools that are available (Figure 1). For relatively new and innovative tools, such as virtual reality and biofeedback, there is a pronounced discrepancy between the perceived value and the perceived skill level with regard to using such a tool is rather high. That is, the perceived value of these tools is relatively high compared with the perceived skill level. This is also the case for tools that involve self-monitoring or a web-based diary. Knowing these discrepancies provides input for designers regarding which eMH tools would fit in a game-based training environment for skill enhancement in eMH. That is, these results help to identify eMH tools that are perceived to be valuable but where lack of skills might hamper their uptake and use in clinical practice. Therefore, these tools are particularly suitable for addressing training environments. In the co-design session, mental health care professionals were provided with more detailed information about the possibilities offered by various eMH tools. After these explanations and examples, the participants in the co-design session perceived the tool to have more value and showed an increased willingness to learn more about these tools compared with their opinions before the session.

#### Users’ Preferences Regarding a Game-Based Training Environment

User preferences for the design of a game-based training environment cover both the general features of the environment and the ways in which the content is being delivered. These preferences were gathered during the interviews and the first co-design session. First, mental health professionals indicated that there should be a clear-cut purpose and that tangible advantages for the user should become instantly clear when engaging in the game environment, as illustrated by the following quote:

In terms of what is in it for me? It may sound stupid, but work pressure is very high, so I don’t have a lot of time for these kinds of things. ... So when I can see that it has a clear purpose and that I will benefit from it in the future; then I would participate.

Respondent 1, psychologist

The game environment should be easily accessible and contain customizable game elements for users with different backgrounds (eg, different levels of adoption, skills, and knowledge) and subsequent training needs. Furthermore, participants indicated that time investment should be done as efficiently as possible, including a smooth transfer of acquired knowledge and skills to their daily practice. This means that it is important to know the actual working situations of mental health care professionals to simulate this as close to reality as possible. Related to this, mental health care professionals stress the need for a game-based training environment in which they can experience the benefits of eMH and practice by building confidence regarding its use in therapeutic practice. A participant stated the following:

If it is offered and I am convinced of its benefits, then I will use it. It should eventually benefit the treatment of my clients, if I can see that it adds to that, then I will more easily use it.

Respondent 5, social worker

Finally, there should be a certain amount of learner control embedded in the game solution, implying that the system should be sufficiently flexible to address different types of needs. This means training for different skill levels, types of skills, flows, and pathways that can be constructed by the users themselves. To satisfy these requirements, it is important to involve end users in the content and design of the environment, as stated by mental health care professionals in this study.

In addition to these preferences regarding the design of the environment, there are also several considerations that mental health care professionals have expressed as to how the content is delivered. The respondents indicated that they were looking for an engaging and playful way of learning with well-defined learning goals. This can be realized by creating a system in which students receive constant and quick feedback and are challenged to take the next step in their learning process. Moreover, the game should be a complete learning experience, with a clear learning goal that is evaluated after playing the game. This is essential for mental health care professionals to transfer from the learning experience to their therapeutic practice. This should also provoke an inductive way of learning. That is, it should support a process of discovering more general rules from specific examples, which enhances the problem-solving capabilities of users. A final need that was mentioned multiple times was the possibility of learning together with colleagues or peers. Making eMH training a team activity would, according to mental health care professionals, be much more effective and engaging than individual training in specific skills. A participant stated the following:

So I think you should make it a group activity and create a good context for using it. … With my team I would take the challenge, but not on my own in my room playing a single player game.

Respondent 13, psychologist

#### User Groups for the Design of a Game-Based Training Environment

Assembling the results on the end users’ needs, 3 main user groups of a game-based training environment can be described. First, a group of mental health care professionals, mainly highly educated nurses, who wanted to learn about the basic possibilities of eMH without putting too much effort and time into it. Training in eMH needs to bring forward positive experiences which in turn could lead to the incorporation of eMH in daily routines. Second, a group of mental health care professionals, nurses, and psychologists, who aimed to bring their eMH skills a step further. These mental health care professionals had already incorporated the basics of eMH in their daily routines but wished to know more about different eMH tools. This group wanted to learn about, for example, the possibilities of dealing with a lack of nonverbal communication in eMH or handling boundaries toward clients. Third, a group of mental health care professionals from various professions, who sought to learn something different. This group of mental health care professionals was eager to try new tools and did not want to be confronted with basic exercises in a game-based training environment. They sought support from team members and wanted to know how to transfer positive experiences when using eMH to colleagues. These 3 main user types reflect the diversity of needs we identified and need to be incorporated into our design through a diversity of suitable scenarios or inherent layering of complexity within a training environment.

### Scenario Development

At the beginning of the Results section, we explained scenario development as an identification of the goals, tasks, actions, and decisions mental health care professionals encounter regarding the use of eMH in daily practice. In this section, we describe the results for these scenarios found in two co-design sessions (sessions 2 and 3; Textbox 1) that were specifically aimed at scenario development.

#### Goals: Purpose of eMH

In the second co-design session, mental health care practitioners were asked to reflect on the purpose of eMH and the steps they deemed important to fulfill this purpose. This led to the description of a catchphrase with regard to the use of eMH in the future: mental health care professionals are familiar and skilled in using innovative treatment solutions, establishing an even better match between mental health services and clients’ needs. The added value of eMH should become clear by demonstrating that web-based treatment can also provide a rich context; for example, it provides mental health care professionals with observable information about someone’s living environment and circumstances. The co-design session also clarified that skill enhancement should enable mental health care professionals to make adequate estimations of the use of different eMH tools in specific situations. Participants stated that knowledge and skills to use eMH should be part of every mental health care professional’s skill set and should be a mandatory competence in every mental health care organization. According to mental health care professionals in the co-design session, eMH has the potential to contribute to much better access to mental health care for many people. For example, a general practitioner can refer clients directly to web-based mental health care without waiting lists.

#### Tasks: Clients’ Characteristics and Therapeutic Situations

According to the survey results and mental health care professionals in the third co-design session, interactions with clients can be aimed at clarifying the problem, finding solutions together, and helping the client handle problematic situations on their own, in other words, knowing how to recognize and cope with different signals with the goal of increasing the client’s symptomatic, personal, and social recovery. In addition, psychoeducation, relapse prevention, and rehabilitation are mentioned as possible therapeutic situations. Whether clients value face-to-face contact with therapists differs among clients. A number of mental health care professionals have reported that some clients would prefer eMH over face-to-face contact, which would even stimulate their engagement and adherence to treatment. This is in line with how the participants in the first co-design session described their work context.

#### Actions: Use of Specific eMH Tools

The range of currently available eMH tools in the practice of mental health care professionals comprises a rather diverse set of tools in terms of maturity and even more so for the level of advancement in training for these tools. The eMH tools that were pointed out in the second and third co-design sessions as worthy of further exploration in a potential game-based training environment are a web-based platform for psychological treatment (consisting of tools such as video calling, web-based modules, and a message function), WhatsApp, self-help programs, web-based group courses, a client portal, web-based screening, virtual reality, and biofeedback (including wearables). This finding is in line with the results of a survey that also elucidated these types of eMH tools. It was emphasized that for mental health care professionals to experience the benefits of using eMH when working with a client, it is important that the type of eMH fits the particular characteristics and needs of the client. In addition, mental health care professionals have stressed the importance of offering eMH tools that are ready to use in practice. Regarding eMH tools that are still in their infancy or experimental stage, a specific group of pioneers can be approached. However, these tools will be less appealing to most mental health care professionals who are still in search of gaining experience with the basic principles of using eMH in general.

#### Decisions: Choice to Use eMH Based on Anticipated Consequences

The nature and complexity of psychological disorders, the client’s age, level of digital literacy, intelligence, and the devices available to the client are factors that are often mentioned by mental health care professionals as having an important influence on the use of eMH in practice. The specific desires and needs of clients regarding the use of eMH tools are something a professional and client need to explore and discover together. In the co-design sessions on scenario development, an important aspect mentioned for the continued use of eMH is that professionals experience is a clear consequence or effect related to the use of eMH in their work. Although there are various treatment trajectories one can think of and various client groups that mental health care professionals see, it is important to create multiple scenarios with different outcomes (ie, different decisive moments to use eMH and perceive the benefits). Moreover, participants in the co-design sessions indicated that it would be valuable if there were several points in a scenario in which participants had multiple options to choose, leading to different experiences of eMH. There needs to be a clear conclusion to the story; for example, that the client is finding a solution to their problem. This should be related to the choices made in the game, and in particular, the choices with regard to using eMH. Providing this conclusion from the perspective of the client within a scenario would, according to the participants of the co-design sessions, significantly contribute to the effectiveness of a game-based training environment.

### Technical and Organizational Prerequisites

In the development of a game-based training environment, it is important to consider the technical and organizational conditions (ie, the prerequisites that need to be met when delivering the final solution). These are crucial for the successful implementation of an envisioned environment within a mental health care organization. Several criteria were identified based on these interviews. A first key aspect elicited in the interviews was the technical support available from their organization when using the environment. This is especially true when offering a technical solution to people who still need to acquire the skills necessary to make more use of technological tools in mental health care in general. Technical issues must be resolved immediately to prevent the risk of negative experiences. A respondent stated this in a straightforward manner:

If it doesn’t work, I am out.

Respondent 3, psychologist

The second aspect is the perceived added value of the training. Mental health care professionals indicated that they had to be convinced of the benefits of game-based training to feel motivated to engage in it. It should therefore be stressed beforehand that using the game-based training environment will eventually lead to better job performance and better treatment for mental health clients. This added value should be communicated, for example, by peer users or educational experts within the organization. Another important factor is management’s push to engage in training. A significant number of mental health care professionals feel that they need some form of obligation to take that step to engage in a game-based training environment. A participant formulated the following:

What should be facilitated by management is a clear assignment. ... That is something that I really miss now that we are a self-managing team. I want to know what space I have and what expectations management has. ... Beginning with defining clear goals for the next year, that is the job of a manager.

Respondent 4, specialized nursing professional

Experiencing a certain lack of skills, and the consequences of that, will trigger a sense of urgency by mental health care professionals. However, the way in which the environment is introduced is crucial. As soon as professionals feel that the management push is solely driven by the goal of saving money and not because professionals or their clients benefit from it, this pressure will elicit a strong feeling of resistance and hamper the adoption of the environment. Therefore, mental health care professionals feel that it is necessary for management to emphasize the added value it can provide. Management support in practical terms is equally important: to enable the effective use of the game-based training environment, time and resources should be made available for mental health care professionals to engage in it. Finally, several professionals mentioned low visibility and awareness of eMH during daily practice as an important influence on the adoption of eMH. Visibility and awareness are also important to consider when developing a game-based training environment. According to the interviewees, external triggers, standard procedures, and good embeddedness in organizational processes are crucial for the adoption of a game-based training environment. A respondent described this as follows:

I think it is an important aspect of the human resource strategy. As an organization you should focus on keeping your employees engaged and loyal. But also embedding this in standard procedures, as in: this is how we work and what we expect of our employees.

Respondent 12, manager

## Discussion

### Principal Findings

In this study, we aimed to identify the user requirements that inform the design of a game-based training environment for mental health care professionals to enhance their knowledge and skills regarding the use of eMH. This has led to specific guidelines for the design of a game-based training environment and has generated multiple ideas regarding its potential content and shape. In addition, the results of this study are useful for gaining a better understanding of mental health care professionals’ attitudes, skills, and ambitions regarding the use of eMH. This can also be used to develop policy interventions to enhance the adoption and implementation of eMH among mental health care professionals and innovation in mental health care.

We identified that the adoption of eMH by mental health care professionals is low and that only 26.2% (113/432) of professionals claim to be active or innovative users of eMH. It is important to note that the survey that generated these results was conducted before the COVID-19 pandemic that started in March 2020, which led to an immediate large-scale (forced) use of eMH, particularly video calling [28]. However, this is not per definition equal to the sustainable adoption of eMH by professionals in the so-called *new normal* in mental health care [29]. In a recent study conducted during the first wave of the COVID-19 crisis, mental health care professionals indicated that the continued use of eMH largely depends on the experienced possibilities of eMH during the COVID-19 period and the context (ie, type of treatment, type of clients and their preferences, and clients’ home situations) in which eMH is applied [29]. At this point in time, sustainable uptake and integration of eMH in the daily practice of mental health care professionals seems to have not yet taken place, despite the fact that many mental health care professionals have gained experience in using eMH under circumstances that require remote treatment [30]. This may be caused by a persistent lack of knowledge and skills regarding the possibilities of eMH or a general conception among mental health care professionals that eMH equals video calling and using web-based modules [31]. There is still relatively little knowledge about different types of eMH [31], and there is still a world to be discovered regarding the possibilities of, for example, virtual reality, biofeedback, and smartphone apps. Furthermore, from the existing literature, we have learned that knowledge and skills consist of different elements; for example, skills to gather information through remote therapy or to create an emphatic interaction [8,13]. This is consistent with the results of our user requirements analysis, which shows that the focus of a training environment for skill enhancement should be aimed at increasing knowledge about the possibilities of eMH in a broader sense, experiencing the benefits in particular situations, and building confidence in using eMH in a therapeutic setting. This requires careful consideration of the game characteristics (eg, player interaction, feedback, and competition) that are suitable for this aim [32,33]. In addition, we determined that there are differences in the characteristics, environment, perceived problems, and preferences of mental health care professionals that should be considered. This means that it is important to address the diversity that exists within the user group and the different requirements that this brings forward regarding a game-based training environment and the way in which it is implemented. This is also mentioned in the literature, where it is discussed that to empathize with users, designers have to know the different user groups [34].

Research into scenarios was aimed at identifying different situations that represent the daily practice of mental health care professionals in which eMH could play a role. For developers of a game-based training environment, it is important that a varied user group can identify the scenarios that are incorporated in the environment [35,36]. For example, mental health care professionals consider various eMH tools valuable. These tools differ in terms of maturity and skills required to being able to use them. Therefore, there is a need to match users’ preferences to the maturity of the tools but also to align the content of a game-based training environment with the required skills and knowledge that users (feel they) are lacking. The results also showed that an important consideration for mental health care professionals to continue using eMH is that a clear and positive outcome is experienced. This means that participants should be allowed to experience the consequence of a certain choice within the game [37], which in this case is the result of using eMH and possible added value. The various treatment trajectories with various client groups indicated that adaptive multilayered scenarios are required to address the practice of the varied group of mental health care professionals.

When developing a training environment, it is crucial to consider how this system will be implemented, that is, the context in which it will be offered and the technical and organizational conditions required [38]. For example, it could be offered by a mental health care organization as an option to support their employees in applying eMH, which could be part of separate vocational training (either optional or mandatory) or incorporated in the curriculum for future mental health care practitioners. Currently, to the best of our knowledge, there are no specific guidelines for training on how to integrate eMH into daily practice; in general, it is not an integral part of the education of mental health care professionals, although several organizations and institutions have started to offer courses on eMH. It is also important to have a clear vision of the demands a mental health care organization sets regarding the intention and skills to use of eMH by its employees and the capacity the organization has to support its employees in meeting these demands. There are several clear expectations that mental health care organizations can have regarding the use of eMH by mental health care professionals (eg, knowing a broad range of possible eMH tools and being able to apply them, a positive attitude toward eMH, and conforming to a new normality). This requires a reconsideration of the ambitions, a smart formulation of the goals the organization has regarding the use of eMH (eg, in the form of formal job requirements) and a plan to support these professionals in acquiring and retaining the active use of the digital possibilities of this era, as well as in mental health care.

### Limitations

The data collection of this user requirements analysis took place in the period before the global pandemic, which may have influenced mental health care professionals’ knowledge and skills regarding the availability of eMH tools in comparison with the results that were collected in this study. However, the use of eMH during the COVID-19 pandemic largely came down to video calling, which means there are many other eMH tools still to be discovered for mental health care professionals [28]. In addition, as becomes clear from current research, there remains a pressing need to invest in training and education of mental health care professionals to effectively use eMH in client-therapist interactions [39].

Another aspect that calls for further exploration is the development of scenarios that more prominently include the client perspective. It should be considered in future research that clients are involved in such design and development processes.

### Conclusions

In this study, we aimed to deliver user requirements to inform the design of a game-based training environment for eMH skill enhancement. To enable a significant improvement in mental health care professionals’ eMH skills, it is important that such an environment is user driven and flexible and simultaneously incorporates the contextual factors that are relevant for its implementation in practice. This user requirements analysis contributes to the understanding of the issues that should be considered in the development of a game-based training environment by showing that “the” mental health care professional does not exist and that a variety of client populations, services, and situations demand a variety of options for training (eg, different difficulty levels and multiple story lines). We used this knowledge for the iterative design process of the serious gaming concept in this project. We designed an eMH escape room with 2 different storylines and explored different eMH tools in different ways. Mental health care professionals were also involved in the design process. These findings can also be of value for other eMH development or implementation projects or for other projects aimed at designing novel learning tools for mental health care professionals in general. Finally, and perhaps even more importantly, this study has provided a very broad and in-depth understanding of what facilitates and hampers the large-scale adoption of eMH in mental health care in general. Therefore, we feel that the results of this study could not only be used as requirements for the design of (technical) solutions for skill enhancement but also as input for policy interventions to stimulate the use of eMH. This can enhance sustainable change regarding the adoption of eMH in mental health care.

## Abbreviations

eMH: e–mental health
GGzE: Stichting Geestelijke Gezondheidszorg Eindhoven en de Kempen
